# Regulatory status of pesticide residues in cannabis: Implications to medical use in neurological diseases

**DOI:** 10.1016/j.crtox.2021.02.007

**Published:** 2021-03-01

**Authors:** Dorina V. Pinkhasova, Laura E. Jameson, Kendra D. Conrow, Michael P. Simeone, Allan Peter Davis, Thomas C. Wiegers, Carolyn J. Mattingly, Maxwell C.K. Leung

**Affiliations:** a School of Mathematical and Natural Sciences, Arizona State University - West Campus, Glendale, AZ 85306, United States; b Pharmacology and Toxicology Program, Arizona State University - West Campus, Glendale, AZ 85306, United States; c ASU Library Data Science and Analytics, Arizona State University, Tempe, AZ 85281, United States; d Department of Biological Sciences, North Carolina State University, Raleigh, NC 27695, United States; e Center for Human Health and the Environment, North Carolina State University, Raleigh, NC 27695, United States

**Keywords:** Cannabis, Pesticide, Database, Regulation, Seizure, Contaminant

## Abstract

Medical cannabis represents a potential route of pesticide exposure to susceptible populations. We compared the qualifying conditions for medical use and pesticide testing requirements of cannabis in 33 states and Washington, D.C. Movement disorders were the most common neurological category of qualifying conditions, including epilepsy, certain symptoms of multiple sclerosis, Parkinson’s Disease, and any cause of symptoms leading to seizures or spasticity. Different approaches of pesticide regulation were implemented in cannabis and cannabis-derived products. Six states imposed the strictest U.S. EPA tolerances (i.e. maximum residue levels) for food commodities on up to 400 pesticidal active ingredients in cannabis, while pesticide testing was optional in three states. Dimethomorph showed the largest variation in action levels, ranging from 0.1 to 60 ppm in 5 states. We evaluated the potential connections between insecticides, cannabinoids, and seizure using the Comparative Toxicogenomics Database. Twenty-two insecticides, two cannabinoids, and 63 genes were associated with 674 computationally generated chemical-gene-phenotype-disease (CGPD) tetramer constructs. Notable functional clusters included oxidation-reduction process (183 CGPD-tetramers), synaptic signaling pathways (151), and neuropeptide hormone activity (46). Cholinergic, dopaminergic, and retrograde endocannabinoid signaling pathways were linked to 10 genetic variants of epilepsy patients. Further research is needed to assess human health risk of cannabinoids and pesticides in support of a national standard for cannabis pesticide regulations.

## Highlights

• Movement disorders are the most common neurological category of qualifying conditions in the U.S.
• The number and action levels of regulated pesticides in cannabis differ vastly in 33 states and Washington, D.C.
• Network analysis reveals potential interactions of insecticides, cannabinoids, and seizure at a functional level.

## 1 Introduction

The pharmacological properties of cannabis and cannabinoids have gained significant research interest in recent years. Cannabis and cannabinoids have been studied for their therapeutic effects in alleviating pain (Hosking and Zajicek, 2008), symptoms related to neurological dysfunction (Devinsky et al., 2014, Watt and Karl, 2017), and psychological conditions such as PTSD (LaFrance et al., 2020). Currently, the U.S. Food and Drugs Administration (U.S. FDA) has approved three cannabis-related and one cannabis-derived drug products (U.S. Food and Drug Administration, 2021). Cesamet, Marinol, and Syndros – the three FDA-approved synthetic cannabinoids – are prescribed for specific use in nausea associated with chemotherapy and anorexia nervosa (U.S. Food and Drug Administration, 2017a, U.S. Food and Drug Administration, 2017b, U.S. Food and Drug Administration, 2006c). Many patients also opt for medical cannabis, which can be easier to access than prescription drugs and has been legalized in more than half of the states in the U.S. (Corroon et al., 2017). However, medical cannabis has not undergone the U.S. FDA approval process, and is not under the same supply chain controls as other prescribed pharmaceuticals.

With the increase in popularity of cannabis and cannabis-derived products, more attention is given to toxicology and human health risk of cannabis contaminants (Dryburgh et al., 2018, Seltenrich, 2019, Seltenrich, 2019, Montoya, 2020). Several cannabis product recalls have been issued in the U.S. due to contamination of insecticides (e.g. abamectin, bifenazate, and malathion) and fungicides (e.g. myclobutanil and tebuconazole) (The Denver Post, 2015, Oklahoma Medical Marijuana Authority, 2020, Oregon Liquor Control Commission, 2021). Additionally, there are reports of pesticide spiking in illegal synthetic products, including brodifacoum (a rodenticide) and paraquat (a herbicide) (U.S. Food and Drug Administration, 2018, Lanaro, 2015). Pesticide use in agricultural commodities is regulated under the Federal Insecticide, Fungicide and Rodenticide Act (FIFRA) (U.S. Government, 1996). Yet, due to the federal status of cannabis as a Schedule I substance (U.S. Drug Enforcement Administration, 2021), the U.S. Environmental Protection Agency (U.S. EPA) has not issued any guideline on pesticide applications in cannabis. Following the wave of legalization of medical or recreational cannabis across the U.S., there is an expectation of the general public that cannabis legalization also results in regulation to ensure safety in cannabis consumption (American Public Health Association, 2014).

In many states, cannabis is recommended by physicians for therapeutic use in various medical conditions. At the same time, there are no federal regulations in place to standardize cannabis as a pharmaceutical. The potential for contamination of cannabis with pesticides is an area of ongoing analysis (Seltenrich, 2019, Stone, 2014, Subritzky et al., 2017, Evoy and Kincl, 2020), and has been observed in medical cannabis samples (Evoy and Kincl, 2020). The inconsistent regulation of medical cannabis, together with potential exposure to harmful pesticides, can result in adverse health outcomes in patients with susceptible conditions. Here, we examine the state-level regulations, publicly available pesticide residue testing reports, and curated biological interactions in the Comparative Toxicogenomics Database (CTD; http://ctdbase.org/; (Davis, et al., 2021) to evaluate the potential neurological hazards of pesticide exposure in medical cannabis.

## 2 Materials and methods

### 2.1 Analyzing state-level regulations of medical cannabis and pesticides residues

We surveyed the online information provided by the public health agencies and agriculture departments of 50 states and Washington, D.C. between September 15 and November 29, 2020. We first determined whether medical and/or recreational cannabis was legalized in each jurisdiction. If medical cannabis was found legal in a jurisdiction, we would categorize the qualifying conditions with reference to the 2017 National Research Council report, “The Health Effects of Cannabis and Cannabinoids”, which described 21 cannabis treatable diseases with different levels of therapeutic evidence (National Research Council, 2017). An earlier study took a similar approach to evaluate the prevalence of qualifying conditions in the U.S. (Boehnke et al., 2019). Here, we mainly focused on neurological diseases in our analysis. We next compared the action levels published by each jurisdiction to regulate pesticide residues in cannabis. If no action level was published online, we would submit a direct inquiry to the cannabis program. We also checked with ISO/IEC 17025-certified laboratories in the state with legalized cannabis (International Organization for Standardization, 2017). With the passage of the 2018 Farm Bill, pesticide applications in hemp are now regulated by the U.S. Department of Agriculture (USDA) under FIFRA (U.S. Government, 1996, U.S. Government, 2018). Thus, we excluded the states that only allowed the use of cannabidiol oil in our analysis.

### 2.2 Evaluating the curated interactions of chemicals, genes, and seizure

We evaluated the potential connections between insecticides, cannabinoids, and seizure using CTD (data release: November 2020, revision 16353). We searched CTD for specific insecticides and cannabinoids to build sets of computational constructed information blocks (i.e., CGPD-tetramers) that related a chemical-gene interaction with a phenotype and seizure, following the methodology previously described (Davis et al., 2020). Briefly, five independently curated data sets (chemical-gene, chemical-phenotype, gene-phenotype, chemical-disease, and gene-disease interactions) were integrated and used as lines of supporting evidence to connect and computationally construct CGPD-tetramers. Each CGPD-tetramer represented a potential chemical-to-seizure connection that met all five lines of evidence. We also compared the gene connections of the insecticide and cannabinoid CGPD-tetramers to the 38 gene variants listed in the 2016 and 2018 reports of the International League Against Epilepsy Genetics Commission (Helbig et al., 2018, Helbig et al., 2016).

### 2.3 Visualizing pesticide regulation and toxicogenomics analysis

We calculated the medians and ranges of pesticide action levels in different jurisdictions. We compared those figures with the tolerances (i.e. maximum residue levels) set for food commodities by the U.S. EPA (U.S. Environmental Protection Agency, 2020) and the reported values of pesticide residues in cannabis. Using Tableau Desktop (version 2020.3.3), we created layered plots that encoded the range of the action levels as gray horizontal lines, and plotted key values as colored circles. In the first chart, the lines served as paths between two values: the minimum and maximum action levels set by each jurisdiction in our data collection. The second chart used a “barbell” style plot, where horizontal lines also served as paths, but these paths connected two different values: the lowest U.S. EPA tolerance levels for food commodities (as adopted by seven states; see Section 3.2) and the median of the action levels. The third chart showed the highest reported values of pesticide residues in cannabis from an open literature search. The action levels, tolerances, and reported values were plotted on a log scale.

Using the CTD CGPD-tetramers, we produced a list of relationships between chemicals and genes, with each relationship weighted by the number of tetramers in the database mentioning the interaction between a chemical and a gene. This produced a weighted edge list that we passed into Gephi, a network analysis and visualization application (version 0.9.2). Using Gephi, we calculated weighted degree centrality, and used the biological functions of genes as node categories. The result was a bimodal network of chemicals × genes, with each gene and their connections to the chemicals color-coded by the gene’s biological function. Functional annotation of the genes used the NIH/NIAID (National Institutes of Health/National Institute of Allergy and Infectious Diseases) Database for Annotation, Visualization and Integrated Discovery (DAVID) version 6.8 (Huang et al., 2009, Huang da et al., 2009). Nodes and edges are sized by weighted degree centrality. Larger nodes indicate chemicals and genes that receive more attention in the CTD curated literature.

## 3 Results

### 3.1 Movement disorders were the most common neurological category of qualifying conditions

We began by surveying the status of cannabis legalization in 50 states and Washington, D.C. Thirty-four states and D.C. permitted cannabis use for medical purpose. Since South Dakota legalized both medical and recreational cannabis on November 3, 2020 (Johnson and Constitutional, 2020, Mentele, 2019), the qualifying conditions for medical use were not yet available. The other 16 states allowed the use of cannabidiol oil only. The medical cannabis programs of 34 jurisdictions (i.e. the 33 states and D.C.) varied greatly in their listing of qualifying conditions (Table 1 and Supplemental Material S1). Three of the jurisdictions had specialized programs for adults and a separate restricted list of qualifying conditions for pediatric use of medical cannabis. Three jurisdictions did not list any explicit condition to qualify medical use. Ten jurisdictions gave physicians full discretion to prescribe outside of the listed conditions. Another 11 jurisdictions allowed petitioning on a case-by-case basis or adding a new qualifying condition at any time, and two jurisdictions allowed public petitioning during legislation changes.

**Table 1:** Quantifying conditions related to neurological dysfunctions, psychological conditions, and pain and injuries for medical use of cannabis in 30 states and Washington, D.C.

Table 1 shows a total of 56 qualifying conditions related to neurological dysfunction (30), psychological conditions (9), and pain and injuries (17) as listed by 31 jurisdictions as well as 2 conditions listed in the NRC report that no jurisdictions explicitly allowed. The average number of enumerated conditions per jurisdiction was 17. One jurisdiction listed as broadly as 53 conditions, while another listed only 9 for non-pediatric prescription. Some of the qualifying conditions were described in language with limited specificity. For instance, many jurisdictions listed “Multiple Sclerosis” as a qualifying condition, but the majority also listed “Severe or Persistent Muscle Spasms”, often in the same sentence. Multiple sclerosis was mentioned together with muscle spasms in 15 jurisdictions. It was mentioned alone in 10 jurisdictions. An additional two jurisdictions listed muscle spasms as a qualifying condition without mentioning multiple sclerosis. Depression and schizophrenia – both of which were reviewed in the 2017 National Research Council report (National Research Council, 2017) – were not listed by any jurisdiction.

**Fig. 1:**
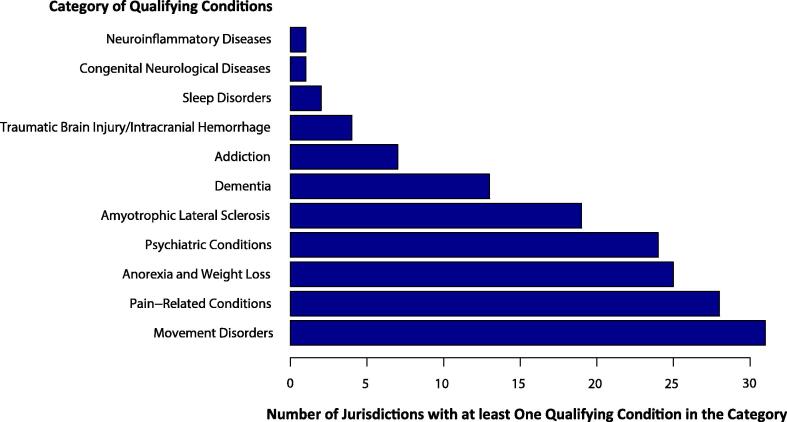
Categories of neurological conditions listed for medical use of cannabis in 30 states and Washington, D.C.

We next examined the listing of 11 neurological categories across the 31 jurisdictions. “Movement Disorders” was the most common neurological category and all 31 jurisdictions listed at least one movement disorder as a qualifying condition (Fig. 1). These conditions included epilepsy, certain symptoms of multiple sclerosis, Parkinson’s Disease, and any cause of symptoms leading to seizures or spasticity. This was consistent with earlier reports that epilepsy and seizure disorders were the two common conditions qualified for medical use in the U.S. (Boehnke et al., 2019, Soroosh, et al., 2020). Based on the language used by these 31 jurisdictions, the authorized use of medical cannabis appeared to be intended to address the movement related symptoms rather than the etiologies of the disorders. “Pain-Related Conditions” was the second most common category (listed by 28 jurisdictions), followed by “Anorexia and Weight Loss” (25) and “Psychiatric Conditions” (24). Many of the qualifying conditions were comorbid such as cachexia/wasting syndrome and HIV/AIDS, cancer, or other causes of major weight loss. Notably, 46 conditions were qualified for medical use by just one jurisdiction (Table 1 and Supplemental Material S1).

### 3.2 Different approaches of pesticide regulation are implemented in cannabis and cannabis-derived products

Medical cannabis is a potential route of pesticide exposure to patients with neurological diseases. Instead of alleviating a patient’s condition, the use of cannabis may harm the patient if it is contaminated by pesticides. We investigated the pesticide testing requirement of cannabis in the state-level jurisdictions with legalized medical use. We found that 24 states and D.C. were posting the pesticide testing requirements and action levels online. We contacted the cannabis programs in the remaining nine states and found that pesticide testing was not required in three states. Also, three states provided no clear response to our inquiries. By the end of this study, we were able to obtain the action levels in 27 states and D.C. In all 28 jurisdictions, pesticide testing of cannabis was required at both the raw agricultural commodity level and the final product level. Six states – Connecticut, Illinois, Louisiana, Maine, North Dakota, and Ohio – adopted the U.S. EPA tolerances for food commodities as the action levels of pesticide residues in cannabis (U.S. Environmental Protection Agency, 2020, State of Illinosis, 2015, State of Connecticut, 2012, Louisiana Department of Agriculture and Forestry, 2017, State of North Dakota, 2019, State of Ohio, 2020). In these states, a cannabis sample would pass the pesticide residue test if it satisfied the most stringent tolerance levels for up to 400 pesticides. Maine also banned the use of 195 pesticides in cannabis that were federally prohibited for use on organic produce (U.S. Department of Agriculture, 2012, Maine Department of Administrative and Financial Services, 2020). Minnesota adopted the pesticide testing guideline for articles of botanical origin provided by the U.S. Pharmacopeia Convention (U.S. Pharmacopeia Convention, 2019). Twenty states and D.C. took a different approach to assess each pesticide and develop action levels individually.

### 3.3 The number and action levels of regulated pesticides differ vastly among jurisdictions

**Fig. 2:**
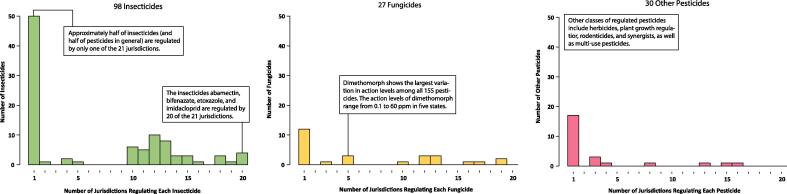
Histograms of 98 insecticides, 27 fungicides, and 30 other pesticides that show how many jurisdictions regulate each of the 155 pesticides in 20 states and Washington, D.C.

Pesticide exposure can result in adverse neurological effects in humans. For instance, acute poisoning of organophosphate and carbamate insecticides results in cholinergic symptoms (i.e. salivation, lacrimation, urination, and diarrhea; or SLUD). We reviewed the 155 pesticides regulated by the 20 states and D.C. (Fig. 2). Insecticides (98) and fungicides (27) were the most two regulated classes of pesticides, followed by plant growth regulators (8), herbicides (5), and rodenticides (4). These 155 pesticides also included 16 organophosphate and 8 N-methyl carbamate insecticides listed in the 2006 and 2007 U.S. EPA reports on cumulative risk assessment (U.S. Environmental Protection Agency, 2006, U.S. Environmental Protection Agency, 2007). The large number of insecticides and fungicides under regulation reflected the industrial practice of using chemical measures to control mite infestation and powdery mildew (McPartland et al., 2000, Punja, 2018). Most of these 21 jurisdictions had action levels for 40–60 pesticides. Abamectin, bifenazate, etoxazole, and imidacloprid were regulated by 20 of the 21 jurisdictions. These four pesticides were also regulated by the six states that adapted the U.S. EPA tolerances. In contrast, 84 pesticides were regulated in only one of the 21 jurisdictions with specified action levels, and only 17 of those were also covered by the U.S. EPA tolerances for food commodities. Lastly, the 155 pesticides regulated by the 20 states and D.C. did not include a number of pesticides previously found in illegal samples, such as brodifacoum, naphthalene, and paraquat (U.S. Food and Drug Administration, 2018, Lanaro, 2015, Fucci, 2003).

**Fig. 3:**
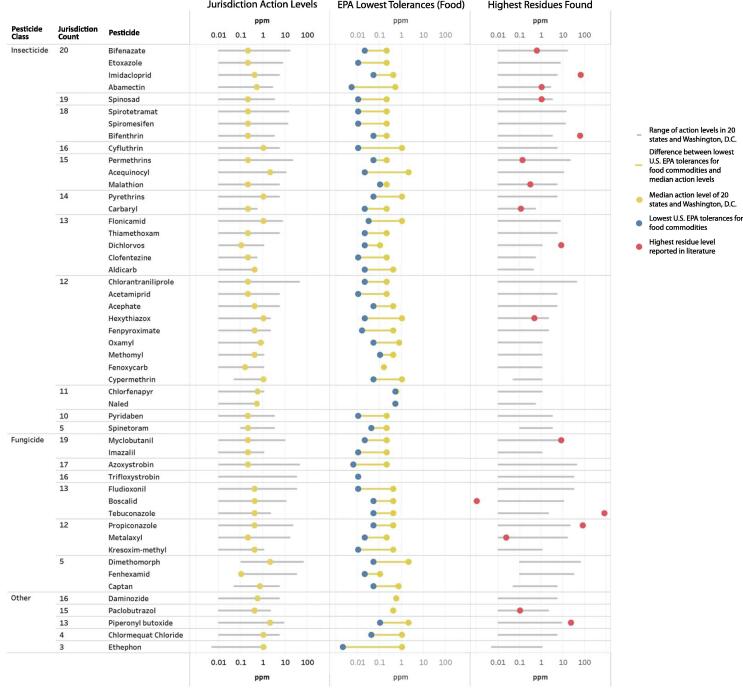
Barbell plots of the top 50 pesticides with the largest variation of action levels in 20 states and Washington, D.C. The blue dots indicated the lowest U.S. EPA tolerances for food commodities, which are adopted for cannabis and cannabis-derived products in seven states. The red dots indicated the highest values of pesticide residues in cannabis plant matter as reported in the open literature (Supplemental Material S2). (For interpretation of the references to color in this figure legend, the reader is referred to the web version of this article.)

Fig. 3 shows the top 50 pesticides with the largest variation of action levels in 20 states and D.C. On average, the action levels of these 50 pesticides were 32-fold higher than the most stringent tolerances for food commodities by the U.S. EPA (U.S. Environmental Protection Agency, 2020). Sixteen out of the 17 reported values of pesticide residues in cannabis plant matter were above the U.S. EPA tolerances for food commodities (Supplemental Material S2). Dimethomorph, a fungicide, showed the largest variation in the action levels, ranging from 0.1 to 60 ppm in 5 states. Azoxystrobin (a fungicide) and chlorantraniliprole (an insecticide) both showed a 4,000-fold difference in action levels. The action levels of these two pesticides ranged from 0.01 to 40 ppm in 17 and 12 jurisdictions, respectively. Ethephon, a plant growth regulator, was regulated by nine states for applications in cannabis. Six of these nine states adopted the U.S. EPA tolerance at 0.002 ppm (for eggs). Two states set their action levels at 1 ppm. The remaining state set its action level at 0 ppm (i.e. zero tolerance) with a target limit of quantitation of 0.005 ppm. In this state, the laboratories were required to detect at least 0.005 ppm of ethephon using their analytical instrument. If their instrument allowed them to detect smaller quantities of ethephon, any amount detected would cause the sample to fail the testing process. California and Florida had two different sets of action levels for inhalable and non-inhalable products (State of California, 2020, Florida Administrative Register, 2020) and Montana also had two sets for unprocessed products and extracts (Montana Administrative Register, 2020).

### 3.4 Network analysis reveals potential interactions of insecticides, cannabinoids, and seizure at a functional level

**Fig. 4:**
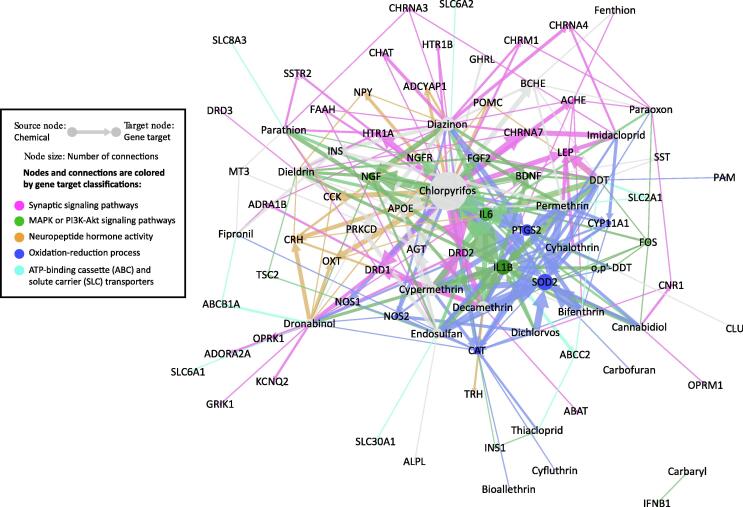
Network view of 621 insecticide and 53 cannabinoid CGPD-tetramers in the Comparative Toxicogenomics Database (CTD). CGPD-tetramers are sets of computational constructed information blocks that related a chemical-gene interaction with a phenotype and seizure as previously described (Davis, et al., 2020). Edges are colored by the biological functions of target genes. The size of nodes indicates the level of attention received by the chemicals and genes in the CTD curated literature.

Comparative Toxicogenomics Database is a powerful tool to identify the potential mechanistic connections between environmental exposure and adverse health outcomes (Davis et al., 2021). We identified 22 insecticides in CTD – including 7 pyrethroids, 6 organophosphates, 4 organochlorines, 2 carbamates, 2 neonicotinoids, and fipronil – and their association with 57 genes, 146 phenotypes, and the outcome of “Seizures” (MESH:012640) in 621 computationally generated CGPD-tetramer constructs (Supplemental Material S3). Chlorpyrifos had the highest number of tetramers (179), followed by diazinon (60) and cypermethrin (52). Only two cannabinoids – cannabidiol and dronabinol – had curated information in CTD. Dronabinol was a synthetic form of Δ-9-tetrahydrocannabinol (THC) approved by U.S. Food and Drug Administration (U.S. FDA) for the treatment of anorexia, nausea, and vomiting associated with AIDS and cancer chemotherapy (U.S. Food and Drug Administration, 2017b, Pertwee, 2012). It was used as a surrogate to highlight the THC-related bioactivity in this network analysis. We further generated 53 CGPD-tetramers with cannabidiol, dronabinol, and seizure and identified 25 genes and 23 phenotypes (Supplementary Material S3). Nineteen genes and 9 phenotypes had connections to both cannabinoid and insecticide CGPD-tetramers. The finding of shared genes and phenotypes was consistent with the fact that many anticonvulsant drugs and insecticides either worked through the same mechanism (e.g. cholinesterase inhibition) or belonged to the same chemical class (e.g. carbamates) (Hen et al., 2012, Kulig and Malawska, 2007, Aracava, 2009). Fig. 4 shows the 246 chemical-gene interactions involved in forming the 621 CGPD-tetramers related to pesticides and seizure. The following are the notable functional clusters in the network:

1. Synaptic signaling pathways, including cholinergic synaptic signaling: ACHE, CHAT, CHRM1, CHRNA3, CHRNA4, CHRNA7, and KCNQ2; dopaminergic synaptic signaling: DRD1, DRD2, and DRD3; and retrograde endocannabinoid signaling: CNR1 and FAAH

2. MAPK signaling pathway: BDNF, FGF2, FOS, IL1B, and NGF; and PI3K-Akt signaling pathway: IFNB1, IL6, INS1, NGFR, and TSC2

3. Neuropeptide hormone activity: ADCYAP1, CCK, CRH, NPY, POMC, OXT, and TRH

4. Oxidation-reduction process: CAT, CYP11A1, NOS1, NOS2, PAM, PTGS2, and SOD2

5. ATP-binding cassette (ABC) and solute carrier (SLC) transporters: ABCB1A, ABCC2, SLC2A1, SLC6A1, SLC6A2, SLC8A3, and SLC30A1

Medical cannabis can potentially expose populations with genetic susceptibilities to harmful contaminants. As a proof of concept, we compared the gene connections of insecticides, cannabinoids, and epilepsy across key biological functions related to seizure (Table 2). Oxidation-reduction process was connected to 18 insecticides, 7 genes, and 183 CGPD-tetramers. It was followed by cholinergic signaling pathway (with 10 insecticides, 9 genes, and 45 CGPD-tetramers) and neuropeptide hormone activity (with 7 insecticides, 7 genes, and 46 CGPD-tetramers). Notably, 10 out of the 38 gene variants in epilepsy patients were related to synaptic signaling pathways, including cholinergic synaptic signaling (CHRNA4, KCNQ1, KCNQ2, and KCNQ3), dopaminergic synaptic signaling (GRIN2A, GRIN2B, and SCN1A), and GABAergic synaptic signaling (GABRA1, GABRB3, and GABRG2) (Helbig et al., 2018, Helbig et al., 2016). Two gene variants – CHRNA4 and SLC2A1 – were connected to 9 CGPD-tetramers with five insecticides (DDT, diazinon, imidacloprid, paraoxon, and permethrin). A complete list of the gene connections of insecticides, cannabinoids, and epilepsy and their overlapping functions can be found in Supplemental Material S4.

**Table 2:** Number of gene connections to insecticides, cannabinoids, and epilepsy across key biological functions related to seizure in the Comparative Toxicogenomics Database.

	Gene	Insecticide Count	CGPD-Tetramer Count		Epilepsy Gene
Pathway and Gene Name	Symbol		Insecticide	Cannabinoid	
**Oxidation-reduction process**					
catalase	CAT	15	29	1	
superoxide dismutase 2	SOD2	13	67	3	
prostaglandin-endoperoxide synthase 2	PTGS2	9	42	5	
cytochrome P450 family 11 subfamily A member 1	CYP11A1	7	15		
nitric oxide synthase 2	NOS2	5	12	1	
nitric oxide synthase 1	NOS1	2	6	1	
peptidylglycine alpha-amidating monooxygenase	PAM	1	1		
**Cholinergic signaling pathway**					
acetylcholinesterase	ACHE	7	10		
cholinergic receptor nicotinic alpha 7 subunit	CHRNA7	4	15		
cholinergic receptor muscarinic 1	CHRM1	3	5		
cholinergic receptor nicotinic alpha 3 subunit	CHRNA3	3	3		
cholinergic receptor nicotinic alpha 4 subunit	CHRNA4	3	6		x
choline O-acetyltransferase	CHAT	2	4		
potassium voltage-gated channel subfamily Q member 1	KCNQ1	0			x
potassium voltage-gated channel subfamily Q member 2	KCNQ2	0		2	x
potassium voltage-gated channel subfamily Q member 3	KCNQ3	0			x
**Neuropeptide hormone activity**					
corticotropin releasing hormone	CRH	5	10	4	
neuropeptide Y	NPY	3	5		
adenylate cyclase activating polypeptide 1	ADCYAP1	2	5		
cholecystokinin	CCK	2	6	1	
oxytocin/neurophysin I prepropeptide	OXT	2	6	3	
proopiomelanocortin	POMC	2	3	1	
thyrotropin releasing hormone	TRH	1	2		
**Dopaminergic signaling pathway**					
dopamine receptor D2	DRD2	4	25	2	
dopamine receptor D1	DRD1	3	13	3	
dopamine receptor D3	DRD3	1	1		
glutamate ionotropic receptor NMDA type subunit 2A	GRIN2A	0			x
glutamate ionotropic receptor NMDA type subunit 2B	GRIN2B	0			x
sodium voltage-gated channel alpha subunit 1	SCN1A	0			x
**Retrograde endocannabinoid signaling pathway**					
fatty acid amide hydrolase	FAAH	3	4		
cannabinoid receptor 1	CNR1	2	2	2	
gamma-aminobutyric acid type A receptor subunit alpha1	GABRA1	0			x
gamma-aminobutyric acid type A receptor subunit beta3	GABRB3	0			x
gamma-aminobutyric acid type A receptor subunit gamma2	GABRG2	0			x

## 4 Discussion

Medical cannabis, like many pharmaceuticals and herbal medicines, are prone to contamination of metals, fungi, and pesticides during manufacturing and storage processes (Seltenrich, 2019, Seltenrich, 2019, Montoya, 2020, Dao et al., 2018, Genuis et al., 2012, Luo, 2021). While pharmaceutical contaminants are under robust U.S. FDA regulations (U.S. Food and Drug Administration, 2020b, U.S. Food and Drug Administration, 2020a), there is the lack of drug safety regulation of medical cannabis at the federal level. Thus, medical cannabis represents a potentially dangerous route of contaminant exposure to patients with susceptible conditions. Here, we surveyed the different approaches taken by the state-level jurisdictions in the U.S. to regulate medical cannabis and pesticide residues. We show that (1) movement disorders are the most common neurological diseases qualified for medical use; (2) the number and action levels of regulated pesticides show great variation between jurisdictions; and (3) exposure to insecticides and cannabinoids affects the same set of signaling pathways that link to seizure.

In the contemporary cultural environment, cannabis is regarded by users and the society more generally as relatively risk free (Bannigan, 0000). An earlier study found that the representations of cannabis risks on social media forums were limited to concerns about driving and sleep effects (Vannoy, 2019). These “risks” were framed as avoidable and ephemeral drug-induced impairments deriving from improper usage. No evidence of concerns was found about adulterated products as the social media representations naturalized cannabis as intrinsically medicinal. This unproblematic naturalization essentially mystifies the chemically-intensive practices used in legal and illegal cultivation as well as the drug safety concerns of cannabis in medical use. The current study reveals a lack of clarity and consistent language in the listing of neurological diseases qualified for medical use. The culture transition of accepting cannabis as a medicinal plant, together with the ambiguity of regulatory language for medical use, creates a potentially dangerous route of contaminant exposure to populations with existing vulnerability.

The observed variation of pesticide action levels is indicative of the legal and scientific challenges in mitigating the human health risk of pesticide exposure in cannabis use. In the U.S., the pesticide residues of crops and vegetables are regulated under FIFRA (U.S. Government, 1996, U.S. Government, 2018). Yet, the illegal status of cannabis at the federal level means that individual states have to develop their own guidance and regulation. The published action levels reflect a variety of strategies taken by the regulatory agencies to approach this problem. Some agencies have developed specific sets of action levels to account for the differences in pesticide-borne health risks due to the concentration effect of the cannabinoid extraction process (Voelker et al., 2015) and the toxicokinetics of inhalational, dietary, and dermal exposures (Sexton et al., 2016, Shiplo et al., 2016, Sullivan et al., 2013). Other agencies opt to impose more stringent action levels by applying the precautionary principle to mitigate such complex exposure scenarios with multiple risks and knowledge gaps (Martuzzi et al., 2004). Implementing the U.S. EPA tolerances of food commodities in cannabis and cannabis-derived products has the advantage of covering a large number of pesticide residues with relatively protective action levels. Yet, the U.S. EPA tolerances are not developed for commodities that are consumed in the inhalable form. Additionally, the effect of pyrolysis on pesticide residues – including the possibility of the generation of hydrogen cyanide – is largely unknown (Sullivan et al., 2013, Health Canada, 2017).

The current study of CGPD-tetramers highlights several pesticide groups that can disrupt multiple biological pathways. Several of these pathways are implicated in seizure, epilepsy, and other neurotoxic effects. For instance, exposure to organophosphate insecticides, carbamate insecticides, as well as cannabinoids can each be linked to oxidative stress and mitochondrial toxicity (Leung and Meyer, 2019, Slotkin et al., 2007, Hebert-Chatelain et al., 2016). Such oxidative stress and inflammation are linked to temporal lobe epilepsy through the MAPK pathway (Drion et al., 2018). Concomitant exposure to organophosphate insecticides and cannabinoids can also cause developmental neurotoxicity (Leung et al., 2019). These pesticide groups may individually, or additively, produce neurotoxic effects though common mechanisms. For example, exposure to chlorpyrifos, diazinon, and dichlorvos all promotes seizure through cholinergic overstimulation. These organophosphate insecticides have been evaluated by the U.S. EPA as a common mechanism group (CMG) (U.S. Environmental Protection Agency, 2006). The CMG approach may be applied to evaluate a specific group of pesticides in cannabis for cumulative risk assessment.

The present study is the first to examine the potential human health hazards of pesticidal contaminants on medical cannabis users. While previous studies have surveyed different classes of prevalent contaminants in cannabis, this study provides a proof of concept that (1) medical use of cannabis may unintentionally expose susceptible patients to harmful pesticides and (2) pesticidal contaminants, cannabinoids, and gene variants may disrupt the same set of biological functions that link to seizure disorders. A number of knowledge gaps remains to be addressed in order to mitigate pesticide-borne health risks in medical cannabis, including (1) the exposure level of insecticide residues in medical patients; (2) the potential interaction of insecticides and cannabinoids and their adverse effects to human health; and (3) the health risk of cannabis use attributed to pesticide exposure and genetic variation. Such exposure and hazard information is crucial to our understanding of human health risk of cannabinoids and pesticides, which will support a health-protective national standard for cannabis pesticide regulations.

## CRediT authorship contribution statement

**Dorina V. Pinkhasova:** Data curation, Formal analysis, Investigation, Validation, Visualization, Writing - original draft. **Laura E. Jameson:** Data curation, Formal analysis, Validation, Visualization, Writing - original draft. **Kendra D. Conrow:** Data curation, Formal analysis, Validation, Visualization, Writing - original draft. **Michael P. Simeone:** Methodology, Resources, Software, Visualization, Writing - review & editing. **Allan Peter Davis:** Data curation, Formal analysis, Methodology, Software, Writing - review & editing. **Thomas C. Wiegers:** Data curation, Formal analysis, Methodology, Software. **Carolyn J. Mattingly:** Funding acquisition, Supervision, Writing - review & editing. **Maxwell C. K. Leung:** Conceptualization, Formal analysis, Funding acquisition, Project administration, Resources, Supervision, Writing - review & editing.

## Declaration of Competing Interest

The authors declare that they have no known competing financial interests or personal relationships that could have appeared to influence the work reported in this paper.
