# Supplementary material for: Regulatory status of pesticide residues in cannabis: Implications to medical use in neurological diseases
Source: Curr Res Toxicol. 2021 Mar 1;2:140–8. doi: 10.1016/j.crtox.2021.02.007 (PMC8296824; doi:10.1016/j.crtox.2021.02.007)
Supplement: Supplementary data 1 [file mmc1.pdf]

**Supplementary Material S1**. Non-neurological quantifying conditions for medical use of cannabis in 30 states and Washington, D.C.

| Other Qualifying conditions | Number of Jurisdictions /31: |
|---|---|
| **Other:** | **31** |
| Admittance to Hospice Care | 3 |
| AIDS | 29 |
| Any other disability | 3 |
| Autoimmune Disease | 1 |
| Cancer | 29 |
| Chronic Pancreatitis | 1 |
| Chronic Renal Failure Requiring Hemodialysis. | 1 |
| Colitis | 1 |
| Crohn's Disease | 21 |
| Cystic Fibrosis | 1 |
| Decompensated Cirrhosis | 1 |
| Ehlers-Danlos Syndrome | 3 |
| Fibrous Dysplasia | 1 |
| Glaucoma | 27 |
| Hepatitis C | 12 |
| HIV | 29 |
| Hydrocephalus | 2 |
| Hydromyelia | 1 |
| IBS | 7 |
| Inclusion Body Myositis | 1 |
| Lupus | 3 |
| MALS | 1 |
| Muscular Dystrophy | 5 |
| Nail Patella | 2 |
| Neuro-Behcet’s Autoimmune Disease (searched Behcet Syndrome) | 1 |
| Osteoarthritis | 1 |
| Osteogenesis Imperfecta | 1 |
| Polycystic Kidney Disease (PKD) | 1 |
| Post Herpetic Neuralgia | 1 |
| Rare condition or disease that affects less than 200,000 individuals in the U.S., as defined in federal law and this is not adequately managed despite treatment attempts using conventional medications (other than opioids or opiates) or physical interventions | 1 |
| Severe Arthritis/ Rheumatoid | 7 |
| Severe Psoriasis and Psoriatic Arthritis | 1 |
| Sickle Cell Anemia | 5 |
| Sjogren’s Syndrome | 1 |
| Superior Canal Dehiscence Syndrome | 1 |
| Terminal Illness | 10 |
| Ulcerative Colitis | 7 |

**Supplemental Material S2**. References with quantified results of pesticide residue testing on cannabis plant matter in the open literature

We searched the open literature on PubMed for papers with quantified results of pesticide residue testing on cannabis plant matter. There were 6 different papers with testing data on cannabis plants that were found through searching “cannabis contaminants” and subsequent searches combining “cannabis” with chemical terms on the PubMed database [1-6]. One paper was also found through a reference in a review paper on PubMed, though the paper itself was not on PubMed[7]. Two other papers came from previous research done on the subject [8, 9]. Of those 9 total papers, 7 of them contained quantifications of specific residues by weight. Most of the pesticide residue testing in those studies were done in cannabis plant matter, so any results from concentrates were excluded in our analysis. Sixteen of the top 50 chemicals had quantified residues found on cannabis shown in figure 3. Nine of these values were found in legal samples from the United States of America, while the other 7 were found in illegal cannabis from 3 different countries.

| CASRN | Chemical Name | Highest Value (ppb) | Ref. | Highest legal USA value (ppb) | Ref. |
|---|---|---|---|---|---|
| 138261-41-3 | Imidacloprid | 64000 | [7] | 64000 | [7] |
| 11141-17-6 | Azadirachtin | 36000 | [7] | 36000 | [7] |
| 51-03-6 | Piperonyl butoxide | 22700 | [7] | 22700 | [7] |
| 62-73-7 | Dichlorvos | 8058 | [7] | 8058 | [7] |
| 88671-89-0 | Myclobutanil | 8039 | [7] | 8039 | [7] |
| 82657-04-3 | Bifenthrin | 60000 | [8] | 5621 | [7] |
| 71751-41-2 | Abamectin/Avermectin | 1000 | [7] | 1000 | [7] |
| 168316-95-8 | Spinosad/ Spinosyn A/D (added) | 1000 | [1] | 1000 | [1] |
| 134-62-3 | Diethyltoluamide/DEET | 880 | [1] | 880 | [1] |
| 40487-42-1 | Pendimethalin | 380 | [1] | 380 | [1] |
| 121-75-5 | Malathion | 300 | [7] | 300 | [7] |
| 658066-35-4 | Fluopyram | 140 | [1] | 140 | [1] |
| 52645-53-1 | Permethrin | 130 | [7] | 130 | [7] |
| 63-25-2 | Carbaryl | 110 | [7] | 110 | [7] |
| 76738-62-0 | Paclobutrazol | 100 | [7] | 100 | [7] |
| 333-41-5 | Diazinon | 70 | [7] | 70 | [7] |
| 56-72-4 | Coumaphos | 40 | [7] | 40 | [7] |
| 149877-41-8 | Bifenazate | 600 | [4] | 30 | [7] |
| 114-26-1 | Propoxur | 30 | [7] | 30 | [7] |
| 4685-14-7 | Paraquat | 25100000 | [3] | | |
| 107534-96-3 | Tebuconazole | 800000 | [8] | | |

| | | | | | |
|---|---|---|---|---|---|
| 1071-83-6 | Glyphosate | 750000 | [3] | | |
| 60207-90-1 | Propiconazole | 80000 | [8] | | |
| 731-27-1 | Tolylfluanid/DMST | 53000 | [8] | | |
| 24579-73-5 | Propamocarb | 9800 | [8] | | |
| 78587-05-0 | Hexythiazox | 460 | [8] | | |
| 57837-19-1 | Metalaxyl | 23 | [6] | | |
| 67129-08-2 | Metazachlor | 22.6 | [6] | | |
| 188425-85-6 | Boscalid | 10 | [4] | | |
| 175013-18-0 | Pyraclostrobin | 10 | [4] | | |
| 953030-84-7 | Buprofezin | 6.6 | [6] | | |


1. Maguire, W.J., et al., *Comprehensive Determination of Unregulated Pesticide Residues in Oregon Cannabis Flower by Liquid Chromatography Paired with Triple Quadrupole Mass Spectrometry and Gas Chromatography Paired with Triple Quadrupole Mass Spectrometry.* J Agric Food Chem, 2019. **67**(46): p. 12670-12674.
2. Fucci, N., *Growing cannabis with naphthalene in Rome.* Forensic Sci Int, 2003. **138**(1-3): p. 91-3.
3. Lanaro, R., et al., *Determination of herbicides paraquat, glyphosate, and aminomethylphosphonic acid in marijuana samples by capillary electrophoresis.* J Forensic Sci, 2015. **60 Suppl 1**: p. S241-7.
4. Moulins, J.R., et al., *Multiresidue Method of Analysis of Pesticides in Medical Cannabis.* J AOAC Int, 2018. **101**(6): p. 1948-1960.
5. Cuypers, E., et al., *The use of pesticides in Belgian illicit indoor cannabis plantations.* Forensic Sci Int, 2017. **277**: p. 59-65.
6. Daniel, D., F.S. Lopes, and C.L. do Lago, *A sensitive multiresidue method for the determination of pesticides in marijuana by liquid chromatography-tandem mass spectrometry.* J Chromatogr A, 2019. **1603**: p. 231-239.
7. Voelker, R. and M. Holmes, *Pesticide use on cannabis.* Cannabis Safety Institute, 2015: p. 1-19.
8. Schneider, S., R. Bebing, and C. Dauberschmidt, *Detection of pesticides in seized illegal cannabis plants.* Analytical Methods, 2014. **6**(2): p. 515-520.
9. Dahl, J., et al., *LCMS-8040 Application Pesticides in Cannabis*. Undated, Shimadzu Scientic Instruments: Columbia, Maryland. p. 8. Table 4.

**Supplemental Material S4**. Gene connections to insecticides, cannabinoids, and epilepsy across key biological functions related to seizure in the Comparative Toxicogenomics Database

| | Insecticide | Cannabinoid | Epilepsy |
|---|---|---|---|
| Neuroactive ligand-receptor interaction | | | |
| Cholinergic synaptic signaling | ACHE, CHAT, CHRM1, CHRNA3, CHRNA4, and CHRNA7 | KCNQ2 | CHRNA4, KCNQ1, KCNQ2, and KCNQ3 |
| Dopaminergic synaptic signaling | DRD1, DRD2, and DRD3 | DRD1 and DRD2 | GRIN2A, GRIN2B, and SCN1A |
| Retrograde endocannabinoid signaling | CNR1 and FAAH | CNR1 | None |
| GABAergic synaptic signaling | ABAT | None | GABRA1, GABRB3, and GABRG2 |
| MAPK signaling pathway | BDNF, FGF2, FOS, IL1B, and NGF | FOS, IL1B, and NGF | CACNA1H |
| PI3K-Akt signaling pathway | IFNB1, IL6, INS1, NGFR, and TSC2 | IL6 | None |
| Neuropeptide hormone activity | ADCYAP1, CCK, CRH, NPY, OXT, POMC, and TRH | CCK, CRH, OXT, and POMC | None |
| Oxidation-reduction process | CAT, CYP11A1, NOS1, NOS2, PAM, PTGS2, and SOD2 | CAT, NOS1, NOS2, PTGS2, and SOD2 | None |
| ABC and SLC transporters | ABCB1A, ABCC2, SLC2A1, SLC6A2, SLC8A3, and SLC30A1 | ABCB1A and SLC6A1 | SLC2A1 and SLC6A1 |
